# Suspended-Core Microstructured Polymer Optical Fibers and Potential Applications in Sensing

**DOI:** 10.3390/s19163449

**Published:** 2019-08-07

**Authors:** Wanvisa Talataisong, Rand Ismaeel, Martynas Beresna, Gilberto Brambilla

**Affiliations:** 1 Optoelectronics Research Centre, University of Southampton, Southampton SO17 1BJ, UK; 2 National Oceanography Centre, Southampton SO14 3ZH, UK

**Keywords:** suspended-core microstructured optical fiber, MPOFs, 3D printer, direct extruded MPOFs

## Abstract

The study of the fabrication, material selection, and properties of microstructured polymer optical fibers (MPOFs) has long attracted great interest. This ever-increasing interest is due to their wide range of applications, mainly in sensing, including temperature, pressure, chemical, and biological species. This manuscript reviews the manufacturing of MPOFs, including the most recent single-step process involving extrusion from a modified 3D printer. MPOFs sensing applications are then discussed, with a stress on the benefit of using polymers.

## 1. Introduction

Photonics crystal fibers (PCF) or microstructured optical fibers (MOFs) typically include multiple air holes that run longitudinally along the fiber. MOFs have attracted increased interest because of their wide range of unique properties such as ultra-wide single mode operation, tailorable dispersion, high or low nonlinearity, and low-loss guidance in selected spectral regions. Because of these features, MOFs have been widely considered for applications in telecoms, power delivery systems, industrial lasers, environmental monitoring, and medicine/healthcare [1,2,3,4,5].

MOFs are an extremely apt device for environmental sensing because the cavities inside the MOFs form natural spectroscopic cells. By using the interaction between the evanescent field and the fluid in the cavities, the fiber itself can be used as a sensor and long interaction lengths can be achieved. All these combined features enable high sensitivities for a wide range of substances. Suspended-core microstructured optical fibers (SC-MOFs), first introduced in 2001 [6], are MOFs where the core is effectively suspended in air by thin membranes connected to a robust solid jacket. Due to the enhanced overlap of the evanescent field of the mode propagating in the core with gases or liquids in surrounding cavities, the SC-MOF is particularly attractive for applications in sensing [7,8,9].

3D printers have been developed and optimized for printing high quality matters from a variety of materials including polymers, glasses, and metals. The improved control system present in the 3D printer, which include built-in temperature controllers and accurate polymer filament feeding systems, allow for the potential use of 3D printers for fiber drawing. Billet extrusion has been shown as a promising single-step technique to fabricate polymer and soft glass optical fiber preforms [10,11,12,13,14]. In this process, preforms are manufactured by pressing a soft bulk polymer or soft glass billet through a die to create a preform with a complex transverse profile, complementary of that of the die. By using this technique, non-circular holes, large air-filling fractions, and long preform lengths, especially for SC-MOF, can be achieved. However, this technique is limited to the manufacture of the structured preforms and a subsequent drawing process is still required to fabricate MOFs.

Here we present the advantage of using microstructured polymer optical fibers (MPOFs) and grating-based MPOFs for sensing applications, including gases, liquids, temperature, and acoustic traces. The technique used to fabricate MPOFs will be reviewed and the recently improved technique to directly extrude MPOFs using a 3D printer will also be presented. The SC-MPOF is directly extruded using a 3D printer, much faster than any others fiber fabrication technique.

## 2. Fabrication of MPOFs

The fabrication of MOFs from a single material shows significant advantages over conventional core-clad optical fibers, because issues related to thermal and chemical compatibility between different materials are intrinsically decreased.

Besides silica and soft glass-based optical fibers, polymer optical fibers (POFs) are one of the most interesting materials for manufacturing optical fibers. Although the POFs exhibit relatively low transmission compared to silica-based fibers, POFs remain flexible even with a large core diameter. The use of polymers for fabricating the optical fiber has been demonstrated due to their low cost for manufacturing and their mechanical ductility for applications in environments exposed to vibrations. Polymer is also an attractive material for sensing, especially biological and chemical, as it is permeable to gases and can be easily functionalized directly.

POFs can also be fabricated via multiple different techniques, and it is relatively easy to incorporate various dopants into the host material. Therefore, POFs are widely used for local area network transmission and sensing applications. The most commonly used polymer for POFs is polymethylmethacrylate (PMMA) which has a theoretical loss limit of 106 dB/km at λ = 650 nm [15]. Other polymers that have been used for POFs include polycarbonate [16] and polystyrene [17], the former being used in applications that require enhanced thermal stability.

**Figure 1:**
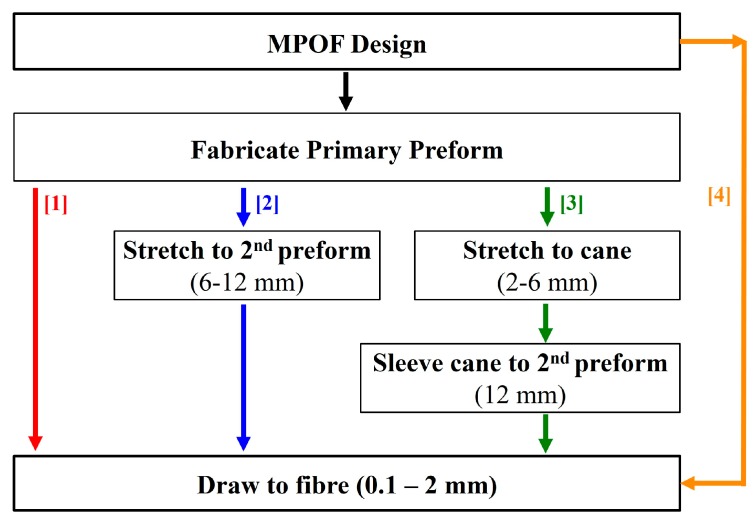
Schematic of microstructured polymer fiber fabrication, showing the three mains fabrication procedures and one new fabrication procedure.

The fabrication of microstructured polymer optical fibers (MPOFs) involves at least two stages and it starts with the manufacturing of a structured fiber preform, which is then drawn into an MPOF, a 2nd preform with a smaller diameter or a fiber cane, using a drawing tower as presented in the schematic of Figure 1. The basic drawing process involves heating the preform to temperatures higher than the glass transition temperature, *T_g_* (around 200 °C for PMMA), to reduce the viscosity of the polymer, and exerting tension to one of the preform extremities, to decrease the diameter. The drawing process often involves intermediate stages, such as the process of drawing a fiber cane [17], which has an intermediate size between the fiber preform and the fiber (diameter ~ 2–6 mm). The cane can be sleeved/jacketed to increase its diameter for specific fiber structures or combined with other canes to form a new preform with even more complex structures.

Polymer is an attractive material for optical fiber devices and sensors because it is deemed suitable for a wider range of fiber fabrication techniques than silica, due to the lower processing temperature and ease of machining. The first microstructured polymer optical fiber preform was demonstrated in 2001 by drilling holes with the desired structure into an extruded PMMA cylinder and then drawing it into an MPOF using a polymer fiber draw tower [18]. MPOF preforms are commonly manufactured using stacking [19], milling [20], polymerization of liquid monomers [21], and casting/molding technique [18,22]. Moreover, there are techniques used to fabricate MPOF preforms for specific structures or applications, such as billet extrusion, used to fabricate preforms with non-circular holes, and rolling of planar stacks, for hollow-core Bragg fibers.

**Figure 2:**
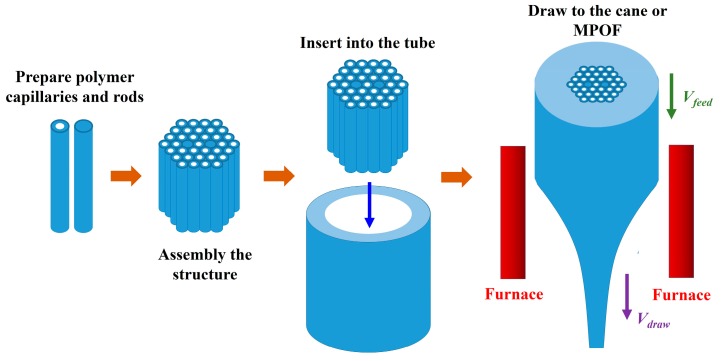
Schematic the stack-and-draw method use to fabricate microstructured polymer optical fibers (MPOFs) and their preform.

The “stack and draw” technique is commonly used to fabricate silica MOFs because it is a versatile and flexible method. By stacking small capillaries, not only various structures can be generated but also different material besides silica glass can be utilized. The preform can be created by assembling rods or capillaries into the desired arrangement and insert the bundle inside a tube to hold the structure securely in place. For MPOF, the polymer rods are inserted into a polymer tube to create an MPOF preform as shown in Figure 2. Before the drawing process, the stacked preform is annealed to remove air and moisture and can affect the final MPOF structure [23]. The advantage of this method is that very large air fractions can be achieved by using a very thin-walled tube. Conventional hexagonal air-hole structures and some complicated superlattice structures can be fabricated using circular capillaries that are arranged in patterns to approximate triangular holes, square holes, and elliptic holes [24,25,26,27,28,29]. Despite this being the most popular method, it is labor-intensive and it is limited to the hexagonal packed periodic structure, thus it is difficult to implement for more complicated designs.

**Figure 3:**
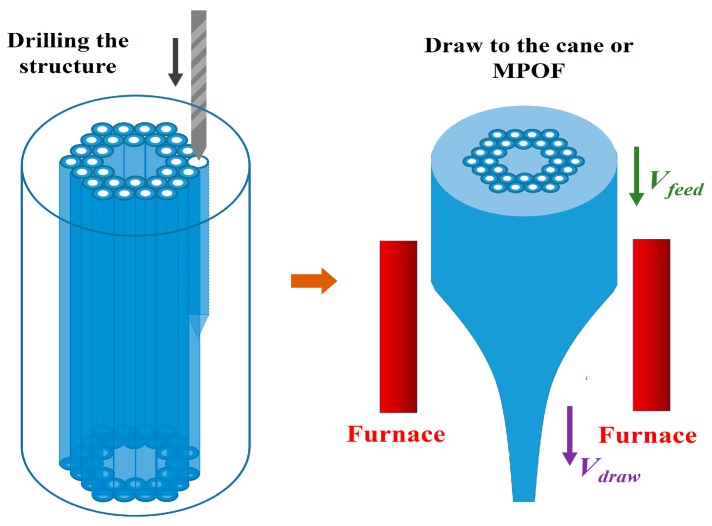
Schematic of the drilling method used to fabricate MPOFs and their preform.

Drilling is a straightforward approach for the fabrication of MPOF preforms. The structure preform is created by drilling the pattern of holes into a solid polymer rod using a drill or a laser (Figure 3) which is connected with an XY translation stage to create the required pattern [30,31,32,33]. Using computer numerical control (CNC) machining it is possible to execute a sequence of automatized instructions over a monolithic polymer cylinder to fabricate complex structured preforms with high resolution. CNC machining offers an easy way to obtain preform with vast varieties of geometries structures by changing the diameters of the holes, their spacing, and distribution in the polymer. Drilling monolithic preforms allows for the rapid prototyping of new MPOF designs. This approach can readily produce structures that are difficult to produce by capillary stacking. However, preform drilling does have many serious limitations, the most common of which is that the process is slow. Moreover, preforms are usually short and large in diameter, typically exceeding 70 mm.

**Figure 4:**
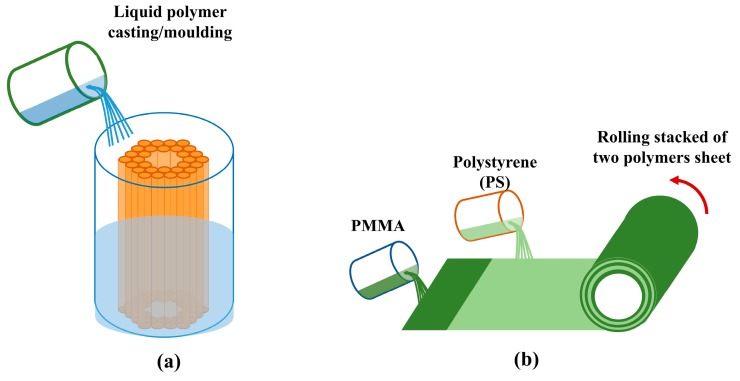
Schematic MPOFs preform fabrication using (**a**) Casting/molding. (**b**) Rolling of planar stack made by solvent deposition.

As far as the MPOF preform rolling technique is concerned, it usually involves two different polymers as presented in Figure 4b. The final preform consists of a hollow-core surround by a dielectric stack made of two different polymers or two materials with different refractive indices. The initial dielectric stack is fabricated in planar form using some form of deposition and it is then rolled into a cylinder to generate the fiber preform. This technique was also commercially exploited to make an Omniguide fiber (Bragg structured fiber [34]) by using a solvent deposition or deposit two polymer (PMMA/Polystyrene) inside of a rotating polymer tube by using solvent-evaporation process. The contrast between the refractive index of different layers generates a Bragg reflector that confines light in the hollow-core [35,36,37,38,39].

Casting is another technology used to produce both glass and polymer preforms [21,22,40]. The necessary chemical precursors (i.e., monomers, initiators and chain-transfer agents) are introduced into a mold to produce the required geometry (Figure 4a). The polymerizing mixture generally requires degassing to avoid bubble formation. After polymerization is complete, the solid structure is removed from the mold leaving only the desired polymer preform structure.

**Figure 5:**
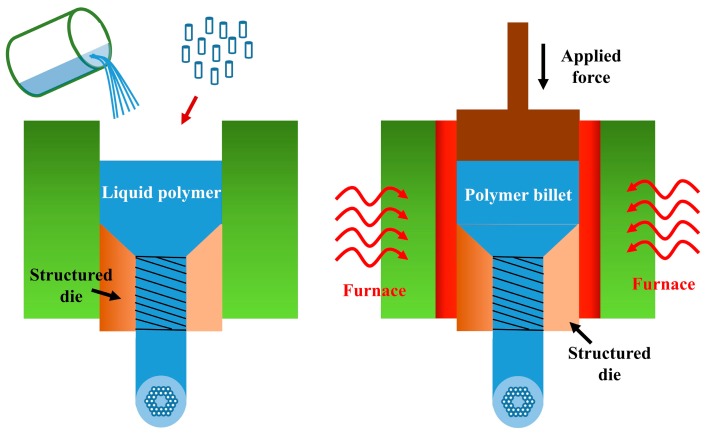
Schematic MPOFs preform fabrication using a liquid polymer extrusion and billet extrusion technique.

The fabrication of an MPOF preform using billet extrusion was first proposed in 2007 [14]. It has been mainly used for compound glasses to produce preforms with complex geometries such as suspended-core and antiresonant fibers [41,42,43,44,45,46,47]. To fabricate the preform, a polymer billet, a monomer, or a resin is forced through a die to form a fiber preform with a complex transverse profile, as shown in Figure 5. One advantage of this method is that it involves a single automated step by which non-circular holes, large air fractions and long preforms can be obtained.

Rapid development of additive manufacturing technologies and the related reduction in cost, moved the fabrication of 3D models into the next generation of high precision manufacturing. 3D printing has drawn a great interest in many fields, such as medicine, art, engineering, and science. Many manufacturing techniques allow to print 3D models, including photo-polymerization, selective laser sintering (SLS), continuous liquid-interface production (CLIP), and fused-deposition modelling (FDM).

In photo-polymerization, a laser or light-emitting diode (LED) is used to scan across a plane and the material polymerization occurs when light interacts with the monomer. To achieve the 3D profile, the laser or LED is controlled by using a three-axial stage and the process is repeated layer by layer. SLS is one of the most popular 3D printing techniques because it can be used to print not only polymer materials but also glass, metal, and ceramic powders. In SLS, a high-power laser is scanned on the horizontal plane layer-by-layer whilst lowering the printing bed. Under the laser irradiation the printing material is fused together forming the desired 3D models. FDM is the most commonly used (and cheapest) technique which relies on the polymer filament fed through a heated nozzle oozing molten polymer. Gluing the molten polymer layer-by-layer generates the 3D profile.

3D printing is widely used for manufacturing optical devices due to its low cost and ease of fabrication. Optical waveguides operating in the telecom, mid-infrared and terahertz spectral regions were the first optical devices fabricated by using 3D printing [48,49,50,51,52,53]. The first hollow-core MPOFs operating in the terahertz (THz) region were 3D printed in 2015 [48]. Due to the long wavelength associated with THz waves, the dimensions of THz fibers are comparable to the resolution of 3D printers, thus, can be directly printed without any further drawing process.

**Figure 6:**
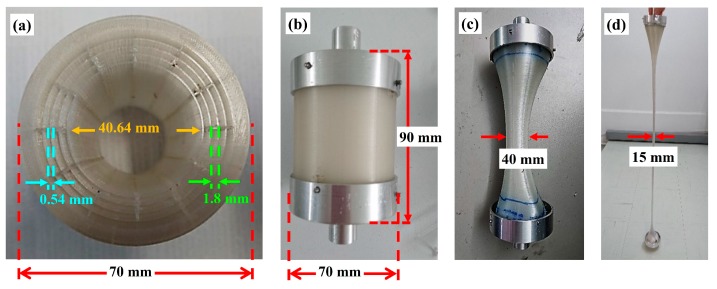
3D printed hollow-core fiber preform for fabricating hollow-core fiber guiding in mid-infrared range [57].

Solid core optical fiber preforms have also been 3D printed and then drawn into fibers [54]. A two-nozzle 3D printer was used to print a step-index fiber preform by using acrylonitrile butadiene styrene (ABS) and polyethylene terephthalate glycol (PETG) filaments. Step-index polymer optical fibers from 3D printed multimaterial fiber preforms was first demonstrated in 2016 [55]. This fiber exhibited the possibility to guide light in telecommunication wavelength region. In 2017, hollow-core fiber (HCF) canes with rectangular and circular shape based on the 3D printed preforms were presented, but no guiding was observed [56]. The first 3D printed hollow-core fiber for the mid-IR guiding was proposed in 2018 (also reported in Figure 6) [57]. In this work, the guidance of fiber in the λ = 3.5–5 µm spectral region was observed.

Drilling and casting are widely used to make MPOFs, but exhibit limitations to the number of transverse features and hole shapes, involve complex steps and require expensive facilities such as clean-room environment for the fabrication [58]. Extrusion seems to be a good technique to fabricate MPOFs preform with non-circular holes, long length, and complex structures. Nevertheless, this technique still requires two steps. To skip the preform preparation, MPOFs can be fabricated in a single step by combining the extrusion technique with the 3D printing technique. The first extrusion of SC-MPOF has been demonstrated in 2018 [59]. The details of nozzle die design and fiber fabrication technique will be presented in Section 3.

## 3. SC-MPOF Extrusion Using 3D Printers

**Figure 7:**
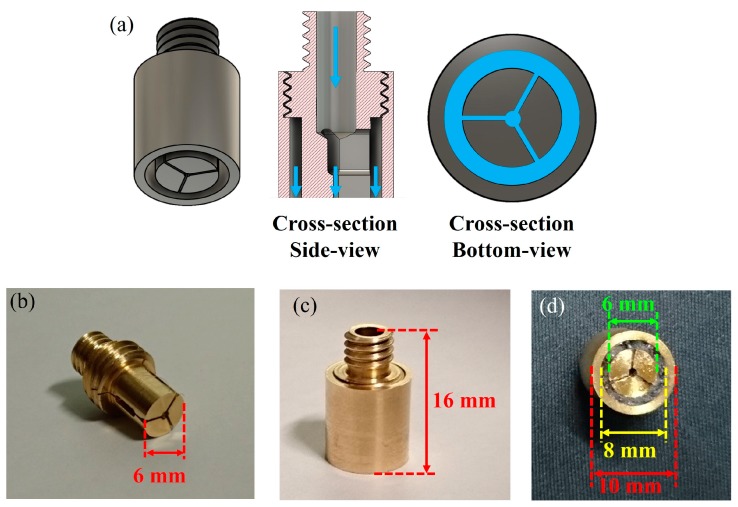
(**a**) Structured nozzle design. From left to right: a 3D model, cross-section of a side- and a bottom-view. Blue arrows represent the direction of polymer flow in the structured 3D printer’s nozzle. (**b**–**d**) Micromachined structured nozzle: (**b**) structured body, (**c**) body + cover, (**d**) after suspended-core (SC)-MPOF drawing [59].

In this work, a desktop fused deposition modeling (FDM) 3D printer was used as an extruder and drawing tower to manufacture the MPOFs. A suspended-core microstructured polymer optical fiber (SC-MPOF) was extruded and directly drawn from a structured 3D printer nozzle. The 3D models of nozzle with an inverse structure profile of the fiber cross-section were designed by using the Fusion 360, Autodesk software. The structured nozzle design was separated into two pieces: the structured body and the cover. While the body shapes the inner structure of the extruded fiber, resulting in the solid core connected by three struts to the outer cladding, the cover determines the outer cladding thickness. The 3D model design was used for CNC machining the nozzle. Indeed, micromachining is a technique traditionally used to fabricate the dies for the extrusion of structured fiber preforms. Figure 7a shows various views of the structured nozzles.

To fabricate SC-MPOFs, the machined structured nozzle was mounted on the 3D printer head while a commercially available 3D printer filament (acrylonitrile butadiene styrene (ABS)) was fed through the heated nozzle. The quality of extruded polymer was tested by extruding the fiber preform through the heated structured nozzle without any additional drawing. The extrusion parameters including feeding speed and nozzle temperature were varied to explore their effect on the visual quality of the extruded fiber preform and achieve the optimal effective temperature experienced by the polymer optical fiber during drawing.

**Figure 8:**
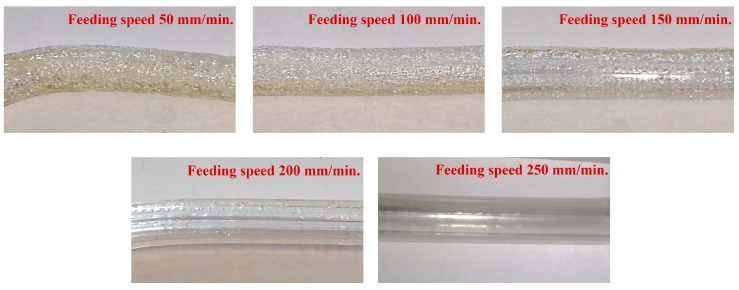
Extruded fiber preform from the 3D printer with different filament feeding speeds at T_n_ = 240 °C.

An initial nozzle temperature T_n_~250 °C was chosen to extruded ABS filament, close to the center of the recommended printing temperature range of 220–270 °C. A filament feeding speed of s~50 mm/min was used for the first try. Air bubbles and a coarse surface were observed in the extruded preform, possibly caused by the high T_n_. These bubbles were attributed to the expansion of air trapped in the filament due to high filament temperature inside the heated nozzle. These bubbles are extremely detrimental as they can result in a deformation of the thin layer and structure inside the fiber and cause the fiber to break during the drawing process. The filament feeding speed also affects the filament temperature: slow speeds result in high filament fictive temperatures, hence affecting the extruded polymer surface quality. The speed was then increased from s~50 mm/min to s~250 mm/min, and an extruded fiber preform with smooth surface was successfully achieved at feeding speed of s~250 mm/min. Surface roughness and defect formation in the extruded polymer optical fiber preform are strongly dependent on the fictive temperature. Nozzle temperature was varied from 230–260 °C to observe the effect of temperature with surface quality of extruded fiber preform. When the nozzle temperature was set to T_n_~240 °C, the smooth and shiny surface were observed and both surface roughness and bubble formation were significantly reduced compared with the other nozzle temperature. So, the temperature of T_n_~240 °C and feeding speed of s~250 mm/min were selected as they provided the best quality of the extruded fiber preform. Figure 8 compares surface roughness of extruded preforms from s~50 mm/min to s~250 mm/min (at fixed T_n_~240 °C).

**Figure 9:**
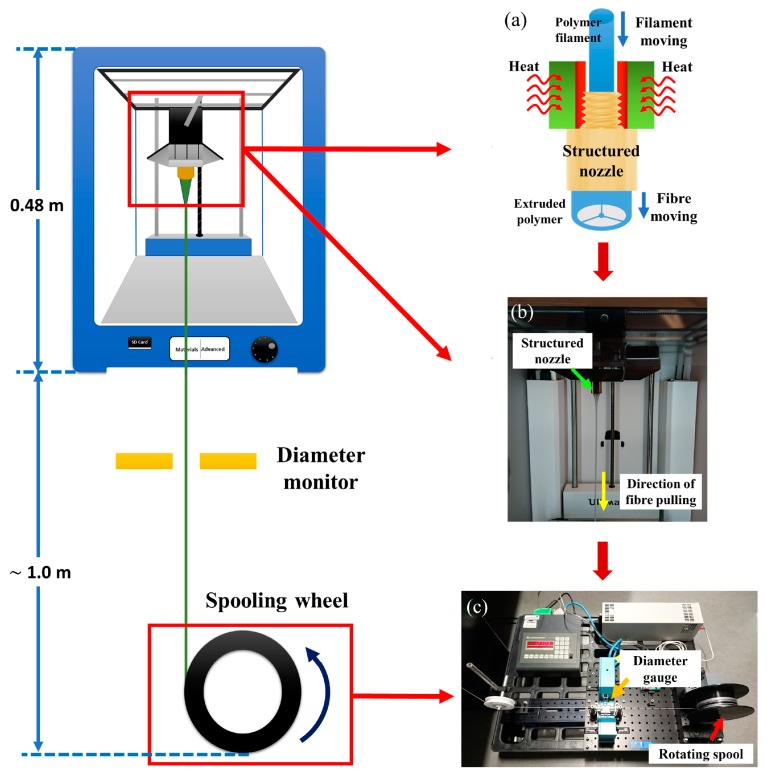
(**a**) Schematic of the experimental setup used to extrude the suspended-core MPOF. (**b**) Fiber drawn from the heated structured nozzle. (**c**) MPOF wrapped onto the 3D printed spool connected to the stepper motor.

An SC-MPOF was extruded at T_n_~240 °C and s~250 mm/min by using a built-in feeding motor and a heater head (Figure 9a,b). An additional processing step, to further reduce the diameter of SC-MPOF, was implemented by connecting the extruded structure to a spool rotating at a constant speed. A stepper motor was used to rotate the spool (diameter of 10 cm) and the fiber drawing speed was controlled by varying the rotation speed of stepper motor. The fiber diameter was monitored in real-time during the drawing process by using an optical diameter gauge (Figure 9c).

**Figure 10:**
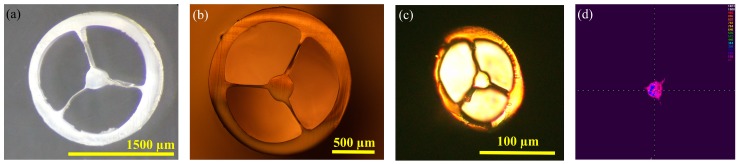
(**a–c**) Microscope images of the fiber cross-section at different diameters. (**d**) Near field image (λ = 1550 nm) at the fiber output for a full turn bending (R~12.5 mm) [59].

The size of the extruded fiber preform prior to drawing is 8 mm, when the preform extrusion rate is 0.58 mm/s at the filament feed speed s~250 mm/min. The SC-MPOF was directly drawn from the heated structured nozzle of the 3D printer with the fiber drawing speed of 58 mm/s resulting in the final diameter (d) drops to d~800 µm. To observe the cross-section of drawn fiber, SC-MPOF was cleaved by using a hot blade. Figure 10a,b show the cross-section of drawn SC-MPOFs with diameters of 1800 µm and 1200 µm after cleaving. The microscope images indicate that the microstructure inside the fiber was maintained after drawing. The surface roughness observed in the extruded preform was further reduced by the fiber drawing and most of the bubbles inside the fiber preform disappeared. The ellipticity observed in the fiber cross-section at small fiber diameters (Figure 10c) has been attributed to the fiber cleaving and, at smaller extent, to the large shear stress exerted by the small diameter rotating spool on the fiber.

## 4. Grating MPOF Based Sensor

A single-mode MPOF is out of interest for communication applications due to the relatively high attenuation of polymers in this wavelength range. However, single mode or few modes MPOFs can be exploited for sensing by inscribing gratings in their cores.

**Figure 11:**
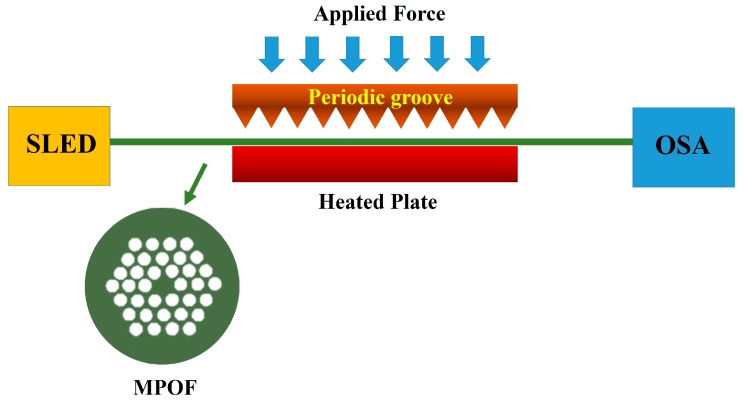
Schematic of mechanical-induced long period gratings (LPGs) in MPOFs.

Long period gratings (LPG) can be easily inscribed by physical imprinting with a periodic pitch of the order of 100 µm to 1 mm. The resonance wavelength associated with the coupling between forward propagating core mode and forward propagating cladding mode in the 500–750 nm range can be achieved with grating periods of 1 mm [60]. Within this region, PMMA-based MPOFs exhibit an extremely low loss, allowing up to 10 m of fiber to be used. Strain [61,62,63], temperature [64], pressure [65], humidity [66,67], and biological sensors [68] using MPOF based LPGs have already been demonstrated. In 2009, a mechanically-induced LPG in an MPOF has been proposed for strain monitoring. A periodic groove with the length and period of 15 mm and 1 mm, respectively, was forced onto the MPOF to create the LPG as shown in Figure 11. The stress was applied to the MPOF based LPG by fixing one end of the fiber while the other was pulled with a rate of 20 mm/min [69]. Another strain sensor using LPG-based MPOF has been revealed in 2012 [70]. The LPG was imprinted into the MPOF using the laser point-by-point technique and a force was subsequently applied to the fiber extremities to induce a strain. A wavelength shift of 20 nm was observed at the maximum applied strain of 4%. A humidity sensor using LPG-based MPOF was demonstrated in 2011 [67]. The laser-inscribed grating had a 1 mm period and was placed in an environmental chamber. The refractive index change due to water absorption was associated with a shift in the resonance wavelength: 10% change of humidity in air could be measured at the temperature of 30 °C.

The polymer biocompatibility was exploited in 2011 to create LPG-based MPOFs for heart rate sensing [71]. Due to the high responsivity of the LPG-based MPOF, a person’s heartbeat can be monitored when the fiber is placed around the torso.

MPOF sensors based on fiber Bragg gratings (FBGs) have also been proposed in single mode and small core multimode MPOF [72] made from PMMA or TOPAS for telecommunication wavelengths. Recently, the thermal response of FBG-based MPOF operating at λ ~ 1560 nm was reported [73] using the flat side MPOF. The shift in the resonance wavelength due to the change of grating period with the applied temperature in the 20–100 °C range was monitored, producing a sensitivity of 95 pm/°C. To benefit from an increased transparency window, FBGs at shorter wavelengths (approaching 800 nm) were demonstrated in 2010 [74]. A grating was inscribed into the MPOF by using a phase mask and a UV laser with λ ~ 325 nm. The inscription of FBG in a multimode MPOF was presented in 2011 [75,76]. These gratings have been deployed for strain and temperature sensing. FBG-based sensors in TOPAS MPOF was also demonstrated in 2011. Due to the low water absorption of TOPAS, this fiber was used for sensing in areas where humidity cross-correlation can be an issue [77]. In 2018, the inscription at short wavelengths of FBG in MPOFs manufactured from various types of polymers (including PMMA, Topas, Zeonex, and Polycarbonate) has been proposed [78]. A pulsed UV KrF laser at 248 nm was used to inscribe a grating operating at the Bragg wavelength of 850 nm. Compared with the 325 nm HeCd laser, the use of KrF laser showed a reduction of the inscription time of at least one order of magnitude.

The advantage of MPOF FBGs and LPGs over their counterparts inscribed in silica fibers is that MPOFs have the largest response to all three stimuli considered, while polymers are biocompatible and extreme a very large ductility and resistance to catastrophic failure. The temperature response of MPOF FBGs and LPGs is 2.5 to 5 larger than that of gratings written silica-based fibers [79,80]. Although the humidity response of PMMA FBGs and LPGs is large [81], this can be eliminated by using TOPAS as material of choice for the fiber fabrication.

In 2011 a comparative study showed that the strain response of FBGs in MPOF is identical to that in step-index when PMMA was used [82]. The few-moded step-index POF had losses approaching 550 dB/m at 700 nm while the single-mode MPOF had a loss <2 dB/m. Compared to FBGs written in silica, the polymer FBGs have a similar strain responsivity, but with a much larger dynamic range (exceeding 20%), and elastic limit (∼4%) [83,84]. Owing to a larger and negative thermo-optic coefficient, FBGs written in polymers have a temperature response 3–4 times larger than those inscribed in silica fibers. The strain response of FBGs and LPGs in MPOF is dominated by changes to the grating period associated with the expansion coefficient, whereas the temperature response in silica is dominated by the thermo-optic coefficient. Polymer can exhibit a strong water absorption, thus a large humidity response, while silica fibers nominally have very little short-term response to humidity. Chirped fiber Bragg gratings inscribed in MPOF was demonstrated in 2018 [85]. The 10 mm-long grating was written by means of a KrF laser and phase mask, and used as a high-sensitivity temperature sensor with a temperature sensitivity of 191.7 pm/°C. A review of polymer fiber Bragg gratings for sensing applications has been reported in 2019 [86], which discussed applications, different material properties, and technique used to inscribe the gratings.

Overall, the potential sensing applications of FBGs and LPGs in MPOF are still being investigated: the large measurable strains, humidity sensitivity, and biocompatibility of polymers can be significant advantages in future applications.

## 5. Liquid and Gas Sensing Based on SC-MOFs and SC-MPOFs

SC-MOFs can be used in nonlinear optics for applications such as supercontinuum generation due to its small field diameter and ruggedness. The small core can also enhance the evanescent field of the propagating mode, thus SC-MOFs have also been developed for sensing applications, especially for gas and liquid detection [6,7,10,87]. SC-MPOFs are often considered a superior technology for biological and chemical sensors owing to their good compatibility with organic chemicals and living cells. They can also be easily transformed into gas and liquid sensors exploiting their intrinsic geometry. These fibers are an attractive platform for liquid sensing because they can enable for strong light-matter interactions, long interaction lengths and the use of small sample volumes. While suspended-core fiber designs are not unique amongst microstructured fibers in providing these properties, they offer the simplest fiber geometry. More specifically, SC-MOFs offer sub-wavelength core dimensions, allowing for large evanescent field, thus light-fluid interaction, and relatively large air holes, allowing for easy filling with gases or liquids.

### 5.1. Dip Sensing

**Figure 12:**
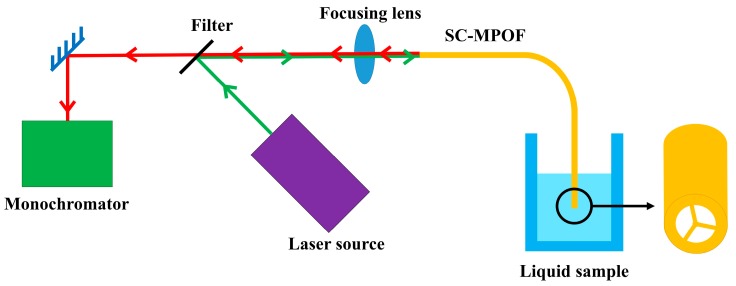
Configuration for SC-MPOF based fluorescence measurement using liquid dipping method.

SC-MPOF can be easily turned into liquid dipping sensor, due to the strong capillarity occurring into the fiber holes. This type of sensor operates by simply dipping one end of the SC-MPOF into a liquid sample (Figure 12). The capillary forces draw the liquid into the fiber holes. Chemical and biological sensors can be easily realized in SC-MPOF by employing functionalization with fluorescent labels on the surface of the fiber core, which are then excited by the evanescent field of the core guided mode. A portion of the fluorescence will be collected by the fiber core and carried through the fiber to a detector (monochromator). Change in the signal of the captured fluorescence are monitored and related to the concentration of the target chemical.

Excitation and fluorescence collection both in the forward and backward guided modes of filled SC-MOF has been studied [88]: the backward collection system provides both the convenience of single-ended access and improvement to the signal-to-pump ratio. In 2009, the fluorescence detection in glass SC-MOF using quantum-dots was demonstrated [89] and provided a detection limit of ~200 pM. Both forward and backward modes were detected by using an optical spectrum analyzer and a photodetector, respectively. The sensitivity of a dipping based SC-MOF quantum dot sensor was then further improved to 10 pM [90] by using a soft glass SC-MOF with a 2 µm core diameter immersed in a suspension containing CdSe quantum dots. A 532 nm laser beam was coupled into the SC-MOF and the fluorescence was delivered to the monochromator after the scattered and reflected pump light were removed with a 533 nm long pass filter. A comparison between the sensitivity of SC-MOF and conventional multi-mode fiber tip sensors has been presented in 2016 [91] showing that the SC-MOF fluorescence sensor has a significantly higher efficiency than the MMF tip sensor.

The use of polymer fibers in fluid sensing can be extremely beneficial because they provide a fast filling time. Since polymers are permeable to fluids, long lengths of fibers can be easily filled in short times without relying on capillarity or cladding drilling.

### 5.2. Surface Functionalisation

**Figure 13:**
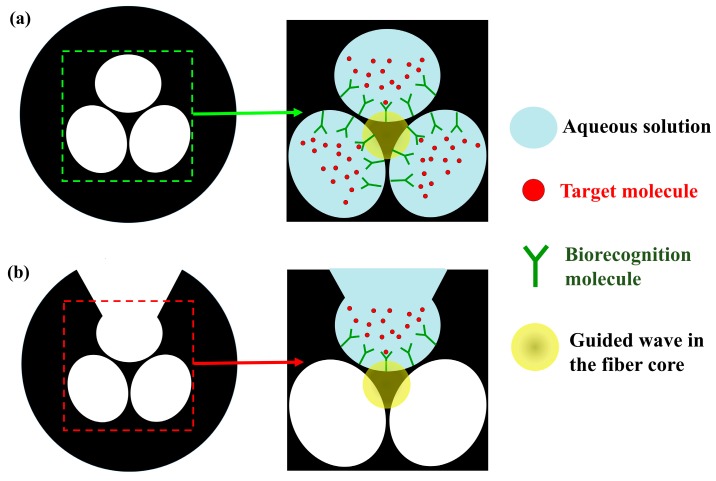
Schematic of surface functionalization for biological and chemical sensing based on (**a**) a conventional SC-MOF, and (**b**) an exposed-core SC-MOF.

Liquid sensors based on SC-MOF using the dipping technique can exploit core surface functionalization with labeling molecules to enhance selectivity and detection limit. There are two main structures of SC-MOF which have been used for biological and chemical sensing: the conventional SC-MOF relies on filling all air holes from one of the fiber ends (Figure 13a) while the exposed-core SC-MOF has a side opening purposely exposed to the environment (Figure 13b). In the former, the functionalization liquid can be delivered through capillarity in the fiber air leaving recognition molecules attached on the surface when the holes are evacuated. In the latter, one of the holes is exposed by laser milling or mechanical micromachining allowing side access the core. The exposed core structure provides an easier configuration to flow fluids through the air holes for functionalization and sensing.

Detection of DNA and antibodies using a functionalized SC-MPOF was first demonstrated in 2006 [92] using a PMMA fiber with 3 holes. DNA strings were fixed by the highly selective hybridization mechanism and the target DNA was captured on the surface of the functionalized fiber core after the hybridization process. A change in the DNA concentration on the surface-induced a change of the refractive index (RI), which was promptly detected. Biomolecules sensing was further developed in 2007 using TOPAS cyclic olefin copolymers (TOPAS COCs) to fabricate a 3 air holes SC-MPOF with 220 µm outer diameter, 50 µm hole diameter, and 12 µm core diameter. Unlike PMMA, which is commonly used for fabricating SC-MOF but has a high-water absorption resulting in bubble formation during the fiber fabrication process, TOPAS COCs has a very low moisture absorption (100 times lower than that of PMMA) [93]. In 2011, aluminum ions were monitored down to a concentration of 100 µM using the SC-MOF [94] fabricated from F2 glass with a core diameter of 1.7 µm and three 12 µm air holes. The coating solution was forced through the fiber holes using a positive pressure of nitrogen gas developed by sealing the extremity of a 6 cm fiber section within a metal chamber with a rubber seal. In 2017, functionalization with plasmonic nanoparticles on a silica SC-MOF with 2 µm core diameter was demonstrated for refractometric sensing [95] over a length of 1–2 cm using different refractive index fluids. A sensitivity of 170 nm/RIU for aqueous analytes was achieved by exploiting various nanoparticle densities ranging over two orders of magnitude. A higher sensitivity of 200 nm/RIU for aqueous analytes at high fringe contrast levels (−20 dB) was observed [96] in exposed-core SC-MOF functionalized with gold nanospheres.

The use of polymer fibers in this field can be extremely attractive because polymers can be easily linked with chemicals used for functionalization, thus do not require the silanization/activation process used to attach the organic molecules used for sensing to the optical fiber core surfaces.

## 6. Physical Sensing Based on SC-MOFs

SC-MOFs based sensors can also be used for a number of physical parameters, such as strain, temperature, pressure, and vibrations.

### 6.1. Strain and Temperature Sensor

**Figure 14:**
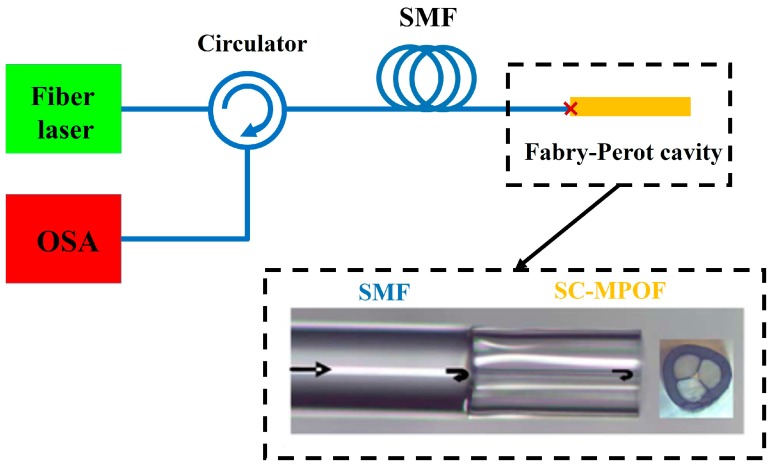
Schematic of experimental setup used for temperature based on Fabry-Perot system using single mode fiber (SMF) spliced with SC-MOF.

A Sagnac interferometer using a section of the SC-MOF as the sensor for temperature-independent strain measurement was presented in 2008 [97]. The sensor was analyzed in two situations, with and without coating. The strain sensitivity of the sensor was observed to be 1.94 pm/με with a very low temperature sensitivity of 0.29 pm/°C. Ge-doped SC-MOF for temperature detection has been demonstrated in 2009 by filling the fiber air holes with a liquid of RI = 1.475 splicing its ends to conventional single mode fibers (SMF) [98]. The temperature change in the 15–35 °C temperature range induced a liquid refractive index change resulting in the shift of the SC-MOF cut-off wavelength. By monitoring the cut-off wavelength shift with the applied temperature, a 25 nm/°C sensitivity was demonstrated. Another SC-MOF Fabry-Perot thermometer has been proposed in 2010 (Figure 14) by splicing unfilled SC-MOF with SMFs and using a dual wavelength Raman fiber laser to generate two quadrature phase-shifted signals that allow the retrieval of the temperature change sensed by the Fabry–Perot interferometric cavity [99].

In 2016, multimode interference was also used in SC-MOF for high temperature sensing [100] up to 1100 °C by measuring the wavelength shifts of the interference pattern. Another thermometer [101] used 20 mm long Bragg gratings created on the core surface by femtosecond laser ablation to monitor temperatures up to 1300 °C. In 2017, a water-filled SC-MOF has been proposed [102] resulting in a sensitivity of water-filled SC-MOF was determined to be 165 pm/°C.

While polymer materials used to manufacture SC-MPOF cannot work at high temperatures because of their intrinsic degradation issues, they exhibit large thermos-optic coefficients (typically an order of magnitude larger than that of silica), large expansion coefficient (one order of magnitude larger) and small Young modulus (three orders of magnitude smaller), thus SC-MPOF sensors can potentially provide sensitivities significantly larger than those observed in sensors based on glass SC-MOF.

### 6.2. Displacement Sensor

In 2012, a micro-displacement measurement based on an SC-MOF Sagnac interferometer was presented [103]. The SC-MOF characterization was made using an optical backscatter reflectometer, screening its multimodal and birefringent behavior. To monitor micro-displacements, stress was induced in the SC-MOF by using two stress inducing plates. Its sensitivity to displacement measurements is shown to be due to birefringence. High precision (∼0.45 μm) was obtained using three different measurement instruments. The advantage of this structure for measuring the displacement is the problem of core-cladding mode coupling can be negligible due to the air cladding.

The use of SC-MPOF could provide a cheaper alternative to the SC-MOF and allow for a significantly larger dynamic range, as polymers typically experience fracture at an elongation one order of magnitude larger than that experienced by silica.

### 6.3. Acoustic Sensor

A fiber-optic acoustic sensor based on SC-MOF was designed and experimentally demonstrated in 2018 [104]. The SC-MOF was spliced to two SMFs to generate a Fabry–Perot interferometer cavity. A compact interrogation system using spectral sideband filtering was constructed for ultrasonic wave detection. The sensor exhibited significantly improved spatial resolution and detection sensitivity by etching the suspended-core diameter to few microns. In the sensing experiment, the ultrasonic wave from PZT was coupled to the SC-MOF. Due to the small fiber core of SC-MOF (<10 µm), the fiber could detect the vibration of air caused by the acoustic wave. Sensor fabrication involved only fiber splicing and etching, which provided a self-shielding cladding surrounding and protecting the core from collisions.

Because of the polymer small Young modulus, SC-MPOF can provide a better acoustic coupling and a larger response to vibrations than silica SC-MOF, thus could potentially give a significantly improved responsivity to acoustic stimuli.

## 7. Conclusions

In the past 2 decade, the development in MPOFs are focused on fabrication techniques and their applications in sensing. Many techniques use to fabricate the MPOFs and their preforms have been reported. A direct drawing of SC-MOFs from a low-cost desktop 3D printer has been demonstrated. This provides the relatively low cost and higher in fabrication speed of current desktop 3D printers compared with a conventional drawing tower, this could become a precious device for the fabrication of microstructured polymer optical fibers. The final fiber cross-section reveals the possibility to maintain the microstructure inside the fiber after the drawing process. Although the fiber size is large compared with commercial optical fibers (Ø ~125 μm), this demonstrates the potential for directly drawing the microstructured polymer optical fiber using a customized 3D printer head.

Sensors based on SC-MOFs have been widely researched over the past ten years and include gas, chemical, biological refractometric, temperature, and vibration sensing. Chemical and biological sensing appear of particular interest due to presence of air holes in the structure. The use of polymers as material of choice for SC-MPOFs can provide significant advantages because of the enhanced permeability to gases, large elongation, large response to acoustic waves, and improved response to temperature. In these fields, future works include permeable fibers, plasmonic and terahertz applications.

MPOFs fabricated from the direct extrusion technique using the 3D printer can provide a cost-effective solution, both in terms of polymer fiber preform and polymer optical fibers, compared to the alternative high-cost and lengthy multi-steps fiber fabrication methods. This breakthrough in manufacturing optical fibers has the potential to transform optical fiber fabrication, and allow emerging new applications, as well as the development of those already in existence, to move to the next generation of sensitivity and dynamic range. This development can be interesting not only in engineering, but also in medical applications, environmental sciences, and academia where the low-cost sensing devices with fast fabrication process are crucial.
